# Proposal of a Preoperative CT-Based Score to Predict the Risk of Clinically Relevant Pancreatic Fistula after Cephalic Pancreatoduodenectomy

**DOI:** 10.3390/medicina57070650

**Published:** 2021-06-24

**Authors:** Marius Lucian Savin, Florin Mihai, Liliana Gheorghe, Corina Lupascu Ursulescu, Dragos Negru, Ana Maria Trofin, Mihai Zabara, Vlad Nutu, Ramona Cadar, Mihaela Blaj, Oana Lovin, Felicia Crumpei, Cristian Lupascu

**Affiliations:** 1 “Gr. T. Popa” University of Medicine and Pharmacy, 700115 Iasi, Romania; marius-lucian.savin@umfiasi.ro (M.L.S.); florin.g.mihai@umfiasi.ro (F.M.); liliana.gheorghe@umfiasi.ro (L.G.); ana-maria.trofin@umfiasi.ro (A.M.T.); mihai-lucian.zabara@umfiasi.ro (M.Z.); nutu.vlad@umfiasi.ro (V.N.); mihaela.blaj@umfiasi.ro (M.B.); cristian.lupascu@umfiasi.ro (C.L.); 2 Department of Radiology, “St. Spiridon” Emergency Hospital, 700111 Iasi, Romania; 3 Department of Surgery, “St. Spiridon” Emergency Hospital, 700111 Iasi, Romania; ramona-petronela-cadar@email.umfiasi.ro (R.C.); felicia.crumpei@umfiasi.ro (F.C.); 4 Department of Anesthesiology and Intensive Care, “St. Spiridon” Emergency Hospital, 700111 Iasi, Romania; oana.apopei@umfiasi.ro

**Keywords:** clinically relevant pancreatic fistula, pancreatic volumetry, computer tomography, pancreatoduodenectomy

## Abstract

*Background and Objectives*: Postoperative pancreatic fistula after cephalic pancreatoduodenectomy (CPD) is still the leading cause of postoperative morbidity, entailing long hospital stay and costs or even death. The aim of this study was to propose the use of morphologic parameters based on a preoperative multisequence computer tomography (CT) scan in predicting the clinically relevant postoperative pancreatic fistula (CRPF) and a risk score based on a multiple regression analysis. *Materials and Methods*: For 78 consecutive patients with CPD, we measured the following parameters on the preoperative CT scans: the density of the pancreas on the unenhanced, arterial, portal and delayed phases; the unenhanced density of the liver; the caliber of the main pancreatic duct (MPD); the preoperatively estimated pancreatic remnant volume (ERPV) and the total pancreatic volume. We assessed the correlation of the parameters with the clinically relevant pancreatic fistula using a univariate analysis and formulated a score using the strongest correlated parameters; the validity of the score was appreciated using logistic regression models and an ROC analysis. *Results*: When comparing the CRPF group (28.2%) to the non-CRPF group, we found significant differences of the values of unenhanced pancreatic density (UPD) (44.09 ± 6.8 HU vs. 50.4 ± 6.31 HU, *p* = 0.008), delayed density of the pancreas (48.67 ± 18.05 HU vs. 61.28 ± 16.55, *p* = 0.045), unenhanced density of the liver (UDL) (44.09 ± 6.8 HU vs. 50.54 ± 6.31 HU, *p* = 0.008), MPD (0.93 ± 0.35 mm vs. 3.14 ± 2.95 mm, *p* = 0.02) and ERPV (46.37 ± 10.39 cm^3^ vs. 34.87 ± 12.35 cm^3^, *p* = 0.01). Based on the odds ratio from the multiple regression analysis and after calculating the optimum cut-off values of the variables, we proposed two scores that both used the MPD and the ERPV and differing in the third variable, either including the UPD or the UDL, producing values for the area under the receiver operating characteristic curve (AUC) of 0.846 (95% CI 0.694–0.941) and 0.774 (95% CI 0.599–0.850), respectively. *Conclusions*: A preoperative CT scan can be a useful tool in predicting the risk of clinically relevant pancreatic fistula.

## 1. Introduction

Despite the many advances in preoperative imaging, refinements of the operative technique and postoperative management, pancreatic fistula (PF) remains the main and feared complication after cephalic pancreatoduodenectomy (CPD), causing high morbidity (i.e., high risk of acute pancreatitis, hemorrhage and severe peritonitis) and a prolonged hospital stay [1,2,3].

Previous studies identified several risk factors for PF, including a soft pancreas (lipomatosis), hard pancreas (fibrosis), Wirsung duct caliber and obesity [4,5].

The consistence of the pancreas is a subjectively assessed intraoperative parameter that is determined by the balance between the lipomatosis and fibrosis and affects the quality of the pancreatoenteric suture [6,7]. Although the pathologic assessment is the most accurate in determining the parameters of the texture of the pancreas, a preoperative multisequence computer tomography (CT) can determine the degree of fatty infiltration of the pancreas and the fibrosis, based on the unenhanced density and the pattern of enhancement of the pancreas [8,9,10,11].

A high caliber of the main pancreatic duct favors a good pancreaticojejunal anastomosis and reduces the risk of PF, provided there is a duct-to-mucosa anastomosis [12].

The pancreatic remnant volume has been proven to independently predict the risk of clinically relevant pancreatic fistula [13].

The aim of the study was to assess the capability of independent parameters evaluated on a preoperative CT scan to predict the clinically relevant pancreatic fistula and to draw up a score based on a logistic regression analysis to detect high-risk patients.

## 2. Materials and Methods

### 2.1. Study Design and Patients

This retrospective study was approved by the Bioethics Committee of “Gr. T. Popa”, University of Medicine Iasi (No.19802, approved date: 14 April 2020). We searched our prospectively maintained database for patients who underwent CPD between 2015 and 2020 that also had an available preoperative multisequence CT scan of the abdomen. The CPD was performed for preoperative high suspicion of pancreatic head cancer or periampullary tumors. No distant metastases or borderline resectable tumors were noted preoperatively. We excluded patients where the marked atrophy of the pancreas did not allow the drawing of a sufficiently large region of interest (ROI) to determine the density of the pancreas and where the pancreatic resection was not performed at the level of the left margin of the superior mesenteric vein (SMV).

All the patients included in the study were represented by randomized numeric codes, and all personal data were hidden.

### 2.2. Surgical Procedure for CPD

The surgical procedure consisted of a backwards Whipple (right posterior superior mesenteric artery—first approach), including a pylorus resection. All the patients underwent a pancreaticojejunal temporarily stented “duct-to-mucosa” end-to-side anastomosis, a hepaticojejunal end-to-side anastomosis and a side-to-side gastrojejunostomy. A standard lymphadenectomy was undertaken in all cases. The procedures were performed by the same surgical team. The mean operating time was about 5.5 h, with a mean blood loss of ~350 mL, consistent with the reported values in other centers [14]. The patients were administered antibiotherapy up to the 3rd day after surgery, as far as no sign of infection was noted. All patients received a single dose of intraoperative Octreotide injection and intravenous H2 receptor antagonists each 12 h throughout the period of absent oral intake. Peritoneal drainage was measured daily. An early postoperative CT scan was performed at days 8–12, followed by the removal of the draining tubes if no fistula or other complications were noticed.

### 2.3. Clinically Relevant Pancreatic Fistula

The pancreatic fistula is defined as a peritoneal drainage at more than 3 days after surgery of any quantity of fluid, with an amylase content of more than 3 times the seric amylase. The 2016 ISGPF classification and grading of pancreatic fistula makes a clear distinction between a biochemical leak (grade A) and clinically relevant fistula (grades B and C) [15]. Grade B pancreatic fistula requires adjusting the clinical management with antibiotherapy, nutritional supplements, Somatostatin analog and percutaneous drainage. Grade C pancreatic fistula can result in sepsis, abdominal hemorrhage, multiorgan failure and even death.

Early postoperative CT images were assessed for the presence of fistulas, seen as fluid collections with or without gas, adjacent to the pancreatojejunal anastomosis [16].

### 2.4. MDCT Acquisition Protocol

For 55 of the 78 patients, the preoperative examination was performed using the 16-slice computed tomography (CT) scanner Somatom Sensation 16 (Siemens Medical Solutions, Erlangen, Germany) at a tube voltage of 140 kV, tube current of 150 mA, standard convolution kernel, mean reconstruction field of view of 37 cm and matrix size of 512 × 512. The scanned volume extended from 2 cm above the diaphragm to the level of the pubic symphysis. Since our institution is a tertiary care center, 23 patients had preoperative CT acquisitions performed in other hospitals using nonstandard dynamic scan protocols. To minimize the effect of different slice thickness and reconstruction filters, we reformatted the images with a slice thickness of 5 mm and an increment of 4 mm, using the diagnostic capabilities of the diagnostic console Intellispace Philips 10 available at our institution.

The preoperative scan was performed after the patients ingested 500 mL of diluted positive-contrast solution to facilitate the assessment of the anastomoses and the visualization of potential collections. When a nasogastric tube was present, it was clamped 1 h before the procedure.

Standard scan protocol included an unenhanced scan and an injection of iodine contrast media at a rate of 3 mL/s with bolus tracker monitorization in the upper abdominal aorta, followed by two scans timed at 15 s and 40 s after the threshold of 150 HU. An additional delayed scan was performed at 3 min after the injection of the contrast in most patients.

### 2.5. Investigated Parameters and Rationale:

Our study investigated the possibility of using imaging-only parameters for the assessment of the risk of clinically relevant pancreatic fistula, objectively quantifiable on widely available preoperative CT scans without taking into account the intraoperative parameters (blood loss, operative time and vascular resections) or disease-specific parameters (TNM staging and neoadjuvant therapy).

Liver steatosis, as a possible indication of associated metabolic disorders, was assessed using the density of the liver during unenhanced preoperative scans. For each patient, we acquired three measurements with a region of interest (ROI) of 1 cm^2^ and noted the mean value.

**Figure 1:**
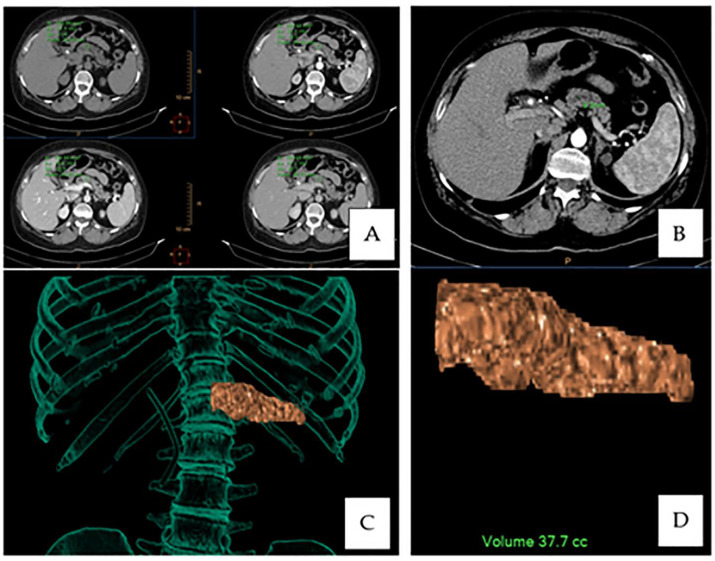
Parameter acquisitions: (**A**) Pancreatic density on a multisequence CT scan at the site of resection. (**B**) Main pancreatic duct measurement. (**C**,**D**) Volumetric rendering overlay and estimated pancreatic remnant volume calculation.

The density of the pancreas is useful both for the evaluation of the degree of lipomatosis and for the identification of the degree of fibrosis. The pancreatic density was measured with a ROI of 1 cm^2^ placed at the estimated level of resection, excluding vascular structures, tumoral tissue, calcifications and ductal ectasias while minimizing partial volume effects from the surrounding structures; three measurements were performed, and we noted the mean value on the unenhanced scans and in the arterial, venous and late scans (Figure 1A). We also investigated the difference between the density of the pancreas in the arterial phase and delayed phase as a possible indicator of fibrosis of the pancreas but were limited by the availability of the scans (not part of the standard protocol) or the inconsistency of the timing of the delayed scans (3, 4 and 10 min).

The caliber of the main pancreatic duct was considered, as it is potentially useful in estimating the quality of the pancreaticojejunal -anastomosis. We noted the caliber of the duct at the estimated site of pancreatic resection (Figure 1B).

An estimated remnant pancreatic volume was considered, as it can potentially influence the risk of pancreatic fistula, patients at a high risk having a greater pancreatic volume, with a greater surface area of the pancreato-entero anastomosis. Volume estimation was performed using the volumetric tools in Intellispace 10 software (Philips) using a clipping plane at the left of the superior mesenteric vein (Figure 1C,D).

### 2.6. Statistical Analysis

The statistical analysis began by inspecting the continuous variables for the normality of distribution using the Shapiro–Wilk test (*p* > 0.05), histogram analysis and z score.

For the analysis of the correlation of the continuous numeric independent parameters with the clinically relevant pancreatic fistula (CRPF), we used a univariate analysis. Statistically significant independent parameters were used in the multiple logistic regression to evaluate the contribution of independent risk factors in the univariate analysis.

The predictive power of the models was analyzed using the area under the receiver operating characteristic curve (AUC) method. A concordance index > 0.8 was considered reliable.

In all statistical tests, a value of *p* < 0.05 was considered statistically significant.

All statistical tests were performed with SPSS version 23 (IBM Corp., Armonk, NY, USA).

To compare the strength of the proposed models, we first verified the correlation with the alternative fistula risk score (aFRS) [17] using Spearman’s correlation and then assessed the difference between them using the DeLong test in MedCalc Statistical software version 20.006 (MedCalc Software Ltd., Ostend, Belgium).

## 3. Results

In total, 78 patients were enrolled in the study: 40 females (51.3%) and 38 males (48.7%), with a mean age of 59.33 years (SD 11.47) and a median body mass index (BMI) of 21 (IQR: 19–23) Kg/m^2^ (Table 1).

Clinically relevant postoperative pancreatic fistula was observed in 22 patients (28.2%). A correlation of the morphological parameters assessed on preoperative CT with the risk of clinically relevant pancreatic fistula is illustrated in Table 2.

A total of five independent parameters were found to be significantly different in the group with CRPF compared to the non-CRPF group: the unenhanced density of the liver (UDL), the unenhanced pancreatic density (UPD), the delayed scan density of the pancreas, the main pancreatic duct (MPD) diameter and the estimated pancreatic remnant volume (EPRV). Due to the unavailability of the delayed scan of the pancreas in all patients and the differences in the scan protocols between different centers (the timing of the delayed scans varied at 3, 4 or 10 min after the injection of contrast, which could potentially alter the validity of the acquired values), we excluded this parameter from further analysis. Additionally, because of the low number of cases, we tried to restrict the model to three parameters.

Next, we verified the independence of the factors by analyzing the Pearson/Spearman’s correlation of the factors, and we observed a correlation between the unenhanced density of the liver and the unenhanced density of the pancreas (r = 0.29, *p* = 0.07) and the main pancreatic duct diameter (r_s_ = 0.14, *p* = 0.37), suggesting the need to exclude one of these parameters.

In the multiple logistic regression, we compared the predictive power of two models by using three factors, using both the MPD and the EPRV and differing in the third independent parameter. The predictive power of the model using the unenhanced pancreatic density preliminary proved to be higher than the one using the unenhanced density of the liver (92.3% compared to 84.6%). Given that marked pancreatic atrophy is frequent in pancreatic head cancers, we proceeded to investigate both models, with the second model potentially being used as an alternative in these patients.

Model 1 (using ERPV, MPD and UPD).

**Figure 2:**
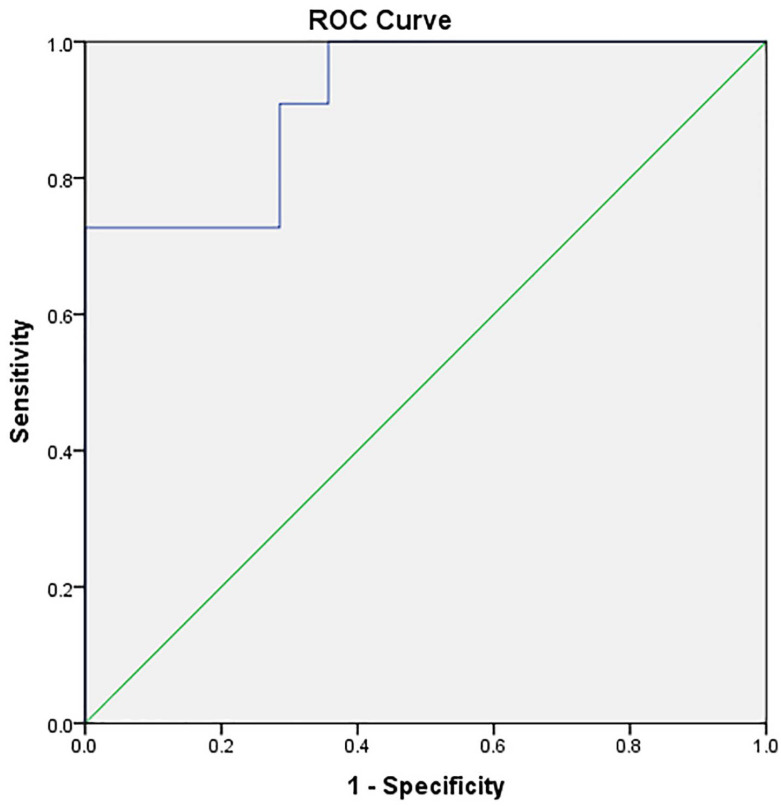
AUC for Model 1 using 3 parameters (preoperatively estimated pancreatic remnant volume, the main pancreatic duct diameter and the values of the unenhanced pancreatic density).

The logistic regression model predicted CRPF after pancreatectomy (Table 3, Figure 2). The risk of CRPF was estimated using the formula y = 1/(1 + e^−z^), where z = −1.114 + 0.155 × (EPRV) − 0.787 × (MPD) − 0.16 × (UPD). A result of > 0.5 estimated a significant risk for CRPF.

To create a practical score, we proceeded to the estimation of the cutoff values for the different parameters. Using the Youden method, we established a cutoff value of 41 cm^3^ for the ERPV (with a sensibility of 81.8% and a specificity of 71.4%) and 30 HU for the unenhanced density of the pancreas (sensibility 90.0% and specificity of 71.4%).

We checked the correlation of the BMI with the unenhanced pancreatic density, as an expression of the metabolic status, using Spearman’s correlation and found a low correlation, although without statistical significance (r_s_ = −0.155, *p* = 0.167). Due to the non-gaussian distribution of the data for the pancreatic duct diameter, we proceeded to estimate the cutoff value for the main pancreatic duct by comparing the AUC of the model using different thresholds, starting from the maximum normal diameter (3 mm) and decreasing by 0.5 mm at each step, obtaining an optimum cutoff value of 2.5 mm.

Based on the odds ratio, we allocated points to the score, as shown in Table 4.

We scored 2.5 points for ERPV higher than 41 cm^3^, 2 points for UPD lower than 30 HU, 1 point for MPD diameter lower than 2.5 mm and 0 points for each of those conditions when they were not met.

**Figure 3:**
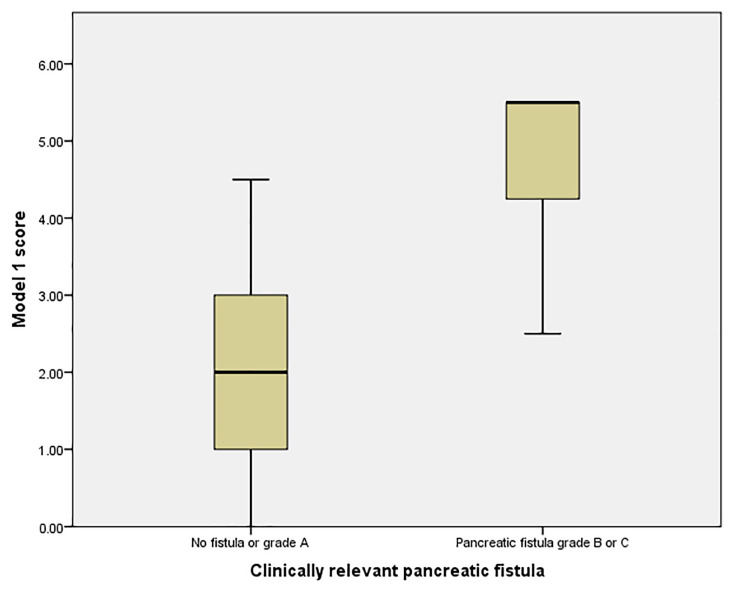
Risk groups for the clinically relevant pancreatic fistula (CRPF) in Model 1.

We analyzed the distribution of the scores related to the clinically relevant pancreatic fistula (Figure 3) and found that a score greater than 3 could identify patients at risk for CRPF with an AUC of 0.92 (95% CI: 0.828–1, sensibility 90.9% and specificity 67.9%) and with a positive predicting value of 47.82% and a negative predicting value of 100%, which suggests the score could correctly identify low-risk patients, although it could not accurately identify high-risk patients.

Using a cutoff score of 4.5 provided a slightly lower AUC of 0.846 (95% CI: 0.694–0.941), sensibility 72.72% and specificity 96.4%) but with a higher positive predictive value (88.88%) and an acceptable negative predictive value (90%), suggesting a good accuracy for the detection of high-risk patients. The mean probability of CRPF for patients with a score of more than or equal to 4.5 was 76.4% (95% CI: 7.35–21.3), compared with 13.84% (95% CI: 58.01–94.92) for scores below 4.5.

**Figure 4:**
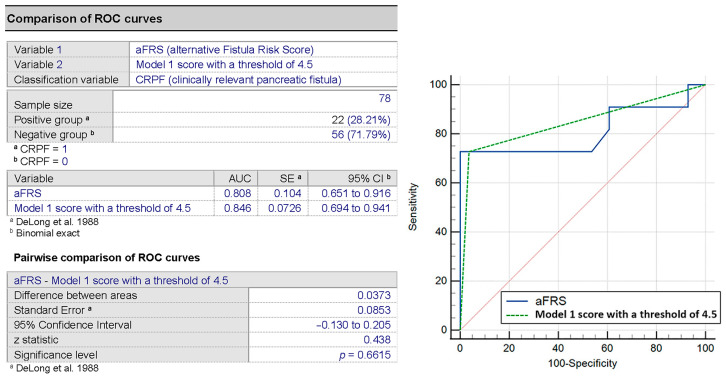
AUC comparison of aFRS and the score in Model 1 for the prediction of CRPF.

A Spearman’s correlation comparison of the score derived from Model 1 to the aFRS showed a good correlation (r_s_ = 0.633, *p* < 0.001). Next, we performed the DeLong test, showing a minor improvement of the AUC using our score compared to the aFRS (AUC 0.846 vs. 0.808) but without being statistically significantly better (*p* = 0.661) (Figure 4).

Model 2 (using ERPV, MPD and UDL).

**Figure 5:**
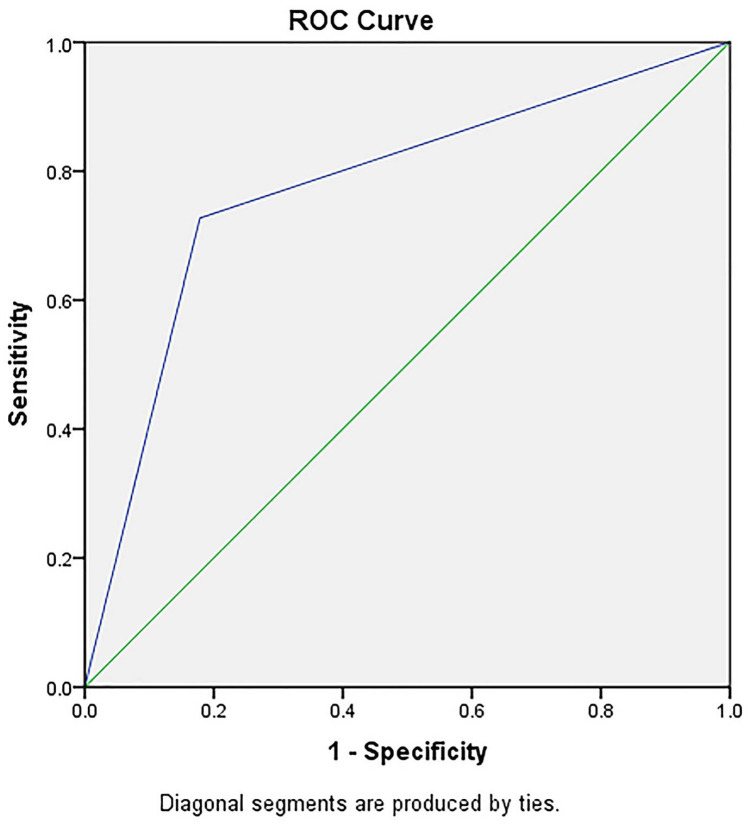
AUC for Model 2 using 3 parameters (the preoperatively estimated pancreatic remnant volume, the main pancreatic duct diameter, the unenhanced liver density).

The risk of CRPF in this model was calculated using the formula y = 1/(1 + e^−z^), where z = 7.219 + 0.138 × (EPRV) − 0.870 × (MPD) − 0.256× (UDL) (Table 5, Figure 5). A result of > 0.5 estimated a significant risk for CRPF.

We calculated the cutoff value for the UDL using the Youden method at 45 HU (sensibility 76.4% and specificity 78.6%). Hepatic steatosis correlated with the BMI with a correlation coefficient r_s_ = −0.130 and *p* = 0.334.

Based on the odds ratio, we allocated points to the alternative score, as shown in Table 6.

In Model 2, we scored 3 points for ERPV higher than 41 cm^3^, 2 points for UDL lower than 45 HU, 1 point for a MPD diameter lower than 2.5 mm and 0 points for each of those conditions when they were not met.

**Figure 6:**
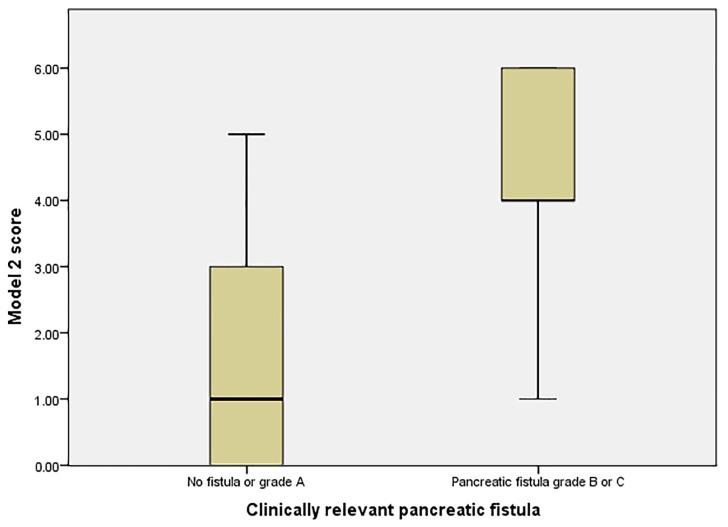
Risk groups for CRPF in Model 2.

A risk stratification analysis (Figure 6) showed that a score greater than 4 produced an AUC of 0.774 (95% CI 0.599–0.850) with a sensibility of 72.7% and a specificity of 82.1%, a positive predictive value of 61.5% and a negative predictive value of 88.46%. The mean probability for CRPF for patients with a score ≥ 4 was 60% (95% CI 37.87–82.13), compared to 12.3% (95% CI 3.79–20.81) for scores < 4.

**Figure 7:**
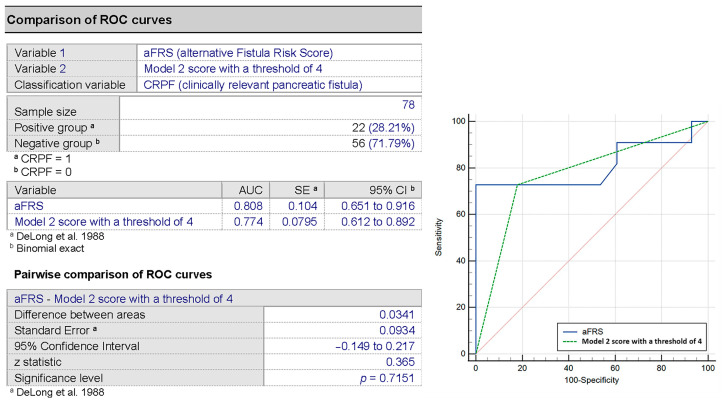
Comparison of the alternative fistula score and our proposed model for the prediction of CRPF.

The correlation of the score model with the aFRS was found to be significant (r_s_ = 0.524, *p* = 0.001). Comparison with the aFRS using the DeLong test showed no improvement over the aFRS (*p* = 0.715) (Figure 7).

## 4. Discussion

Predicting the risk of pancreatic fistula remains controversial, with multiple proposed scores in the literature when using pre-, intra- and postoperative parameters with different accuracy levels (AUC between 0.656 and 0.806) [17,18,19,20,21,22,23].

Although some studies noted the influence of the surgical technique on the risk of pancreatic fistula [24], other authors stated that the type of anastomosis and the placement of the pancreatic stent did not influence the risk of pancreatic fistula [25].

Our study focused solely on evaluating the contribution of independent imaging parameters derived from preoperative contrast-enhanced computer tomography scans and confirmed a number of parameters correlating with the risk of CRPF: the unenhanced density of the liver (the degree of liver steatosis), the unenhanced density of the pancreas (pancreatic steatosis), the delayed density of the pancreas (a parameter that we further excluded because of the inconsistence and nonuniformity in the timing of the sequence between examinations), the main pancreatic duct diameter and the estimated remnant pancreatic volume. An investigation of the correlation of the metabolic status of the patient (expressed through the BMI) with liver/pancreatic steatosis showed only a mild correlation but did not provide statistically significant results, which could be a consequence of both the small sample size and the particularities of the cohort, as multiple patients had a diagnosis of chronic pancreatitis, caused by alcohol consumption, that can alter the measured density of the pancreas [26].

We proposed two scores that can be derived from a preoperatively obtained unenhanced CT scan of the abdomen using the following parameters: the unenhanced density of the pancreas at the estimated site of resection (to the left of the superior mesenteric vein) using a cutoff value of 30 HU, the main pancreatic duct diameter at the estimated site of anastomosis using a cutoff value of 2.5 mm, the estimated remnant pancreatic volume using a cutoff value of 41 cm^3^ and the unenhanced density of the liver using a cutoff value of 45 HU. Although the score using Model 1 (UPD, MPD and ERPV) showed a good performance compared to the aFRS, we derived a second score based on Model 2 (UDL, MPD and ERPV) that did not produce the same performance level but can be a useful alternative in patients with marked atrophy of the pancreas (for whom the confident and reproductible measurement of the unenhanced pancreatic density can be difficult), a frequent situation in pancreatic head neoplasms.

Both scores showed a good sensibility and specificity for the prediction of clinically relevant pancreatic fistula. Although our data showed a mild improvement in the AUC analysis compared to the aFRS in the first model, it did not have a significant improvement when analyzed with the DeLong test, a situation that could be related to the intrinsic limitations of the test when the model was developed and deployed on the same dataset [27].

The good negative predicting value of the scores could be used in the selection of low-risk patients, for whom the complication of mitigation strategies could be adjusted—opting, for example, for a no-drain strategy—especially considering the complications that may occur because of the draining tubes [28,29].

The proposed scores use objective, CT-based independent parameters, irrespective of the intraoperative parameters and histology, and might be an early and useful tool to tailor surgical procedures and postoperative strategies.

The technique employed for the establishment of the cutoff values had a limitation, as they are directly derived from the test set. In our dataset, the threshold value for the pancreatic duct size was found similar to the one found by Xia et al. [19]. Additionally, our study obtained a different cutoff value for the estimated remnant pancreatic volume as compared to Miyamoto et al. [30].

Another shortcoming of our study was the small number of patients, necessitating a further study to validate the proposed CT-based pancreatic fistula risk score.

## 5. Conclusions

Clinically relevant pancreatic fistula can be predicted using preoperative CT scan of the abdomen when using the following parameters: estimated remnant pancreatic volume, main pancreatic duct diameter, pancreatic steatosis and liver steatosis. We formulated two score-based models, the second one having a lower accuracy but useful as an alternative for patients with marked atrophy of the pancreas.

## Figures and Tables

**Table 1:** Baseline characteristics of the retrospective group.

Age, Mean (SD)	59.33 (11.47)	Disease, *n* (%)	
Sex (male), *n* (%)	38 (48.7%)	Pancreatic ductal adenocarcinoma	19 (24.35)
BMI, median (IQR) Kg/m^2^	21 (19–23)	Chronic pancreatitis	7 (8.97)
ASA classification		Neuroendocrine tumor	4 (5.12)
I	18 (23.07%)	Cholangiocarcinoma	6 (7.69)
II	46 (58.97%)	Ampullary carcinoma	24 (30.76)
III-IV	13 (16.66%)	Duodenal carcinoma	12 (15.38)
Comorbidity, *n* (%)		Cystic neoplasms	2 (2.56)
Cardiac	16 (21.79%)	Other	5 (6.41)
Hypertension	17 (21.79%)	Pathology, *n* (%)	
Pulmonary	9 (11.53%)	Malignant	65 (83.33)
Diabetes mellitus	12 (15.38%)	Benign	13 (16.66)

BMI: Body Mass Index; IQR: Interquartile Range; ASA: American Society of Anaesthesiologists Physical Status.

**Table 2:** Univariate analysis of the independent factors and their association with clinically relevant pancreatic fistula (CRPF).

Independent Variable	Non-Clinically Relevant Pancreatic Fistula *N* = 56		Clinically Relevant Pancreatic Fistula *N* = 22		*p*-Value
	Mean	SD	Mean	SD	
Unenhanced density of the liver (HU)	50.54	SD 6.31	44.09	6.80	0.008
Pancreatic density (unenhanced)	32.42	11.93	22.24	11.49	0.02
Pancreatic density (arterial) (HU)	81.35	22.66	73.39	18.63	0.308
Pancreatic density (venous) (HU)	79.23	18.29	67.55	19.66	0.087
Pancreatic density (delayed scan) (HU)	61.28	16.55	48.67	18.05	0.045
Difference between the arterial and delayed density of the pancreas (HU)	19.45	20.32	24.7	15.39	0.42
Main pancreatic duct diameter (mm)	3.146	2.95	0.93	0.35	0.02
Total pancreatic volume (cm^3^)	76.69	31.49	91.31	26.68	0.185
Estimated remnant pancreatic volume (cm^3^)	34.87	12.35	46.37	10.39	0.01

**Table 3:** Multivariate logistic regression for Model 1.

	Weight (β)	Odds Ratio	*p*	CI	
				Lower	Upper
ERPV	0.155	1.168	0.02	1.025	1.330
MPD	−0.787	0.455	0.02	0.235	0.881
UPD	−0.160	0.852	0.032	0.736	0.986
Constant	−1.114				

ERPV: the preoperatively estimated pancreatic remnant volume; MPD: the main pancreatic duct diameter; UPD: the values of unenhanced pancreatic density.

**Table 4:** Proposed score for the assessment of the risk of pancreatic fistula (Model 1).

Variable	Cutoff Value	Weight (β)	OR	Points Allocated	
ERPV (cm^3^)	41 cm^3^	0.155	1.168	If <41 cm^3^ If ≥41 cm^3^	0 points 2.5 points
Unenhanced density of the pancreas (HU)	30 HU	−0.160	0.852	If >30 HU If ≤30 HU	0 points 2 points
MPD diameter (mm)	2.5	−0.787	0.455	If >2.5 mm If ≤2.5 mm	0 points 1 points

ERPV: the preoperatively estimated pancreatic remnant volume; MPD: the main pancreatic duct diameter.

**Table 5:** Multivariate logistic regression for Model 2.

	Weight (β)	Odds Ratio	*p*	CI	
				Lower	Upper
ERPV	0.138	1.148	0.026	1.017	1.295
MPD	−0.870	0.419	0.032	0.189	0.929
ULD	−0.256	0.774	0.032	0.635	0.944
Constant	7.219				

ERPV: the preoperatively estimated pancreatic remnant volume; MPD: the main pancreatic duct diameter; ULD: the unenhanced liver density.

**Table 6:** Alternative score for the assessment of the risk of CRPF using Model 2.

Variable	Cutoff Value	Weight (β)	OR	Points Allocated	
ERPV (cm^3^)	41 cm^3^	0.138	1.148	If <41 cm^3^ If ≥41 cm^3^	0 points 3 points
Unenhanced density of the liver (HU)	45 HU	−0.256	0.774	If >45 HU If ≤45 HU	0 points 2 points
MPD diameter (mm)	2.5	−0.870	0.419	If >2.5 mm If ≤2.5 mm	0 points 1 points

ERPV: the preoperatively estimated pancreatic remnant volume; MPD: the main pancreatic duct.

## Data Availability

The data presented in this study are available on request from the corresponding authors.
